# Traumatic Posterior Atlantoaxial Dislocation Without Associated Fracture but With Neurological Deficit: A Case Report and Literature Review

**DOI:** 10.1097/MD.0000000000001768

**Published:** 2015-10-30

**Authors:** Yong Xu, Feng Li, Hanfeng Guan, Wei Xiong

**Affiliations:** From the Department of Orthopedic Surgery, Tongji Hospital, Tongji Medcial College, Huazhong University of Science and Technology (HUST), Wuhan, China (YX, FL, HG, WX).

## Abstract

Posterior atlantoaxial dislocation without odontoid fracture is extremely rare and often results in fatal spinal cord injury. According to the reported literature, all cases presented mild or no neurologic deficit, with no definite relation to upper spinal cord injury. Little is reported about traumatic posterior atlantoaxial dislocation, with incomplete quadriplegia associated with a spinal cord injury.

We present a case of posterior atlantoaxial dislocation without associated fracture, but with quadriplegia, and accompanying epidural hematoma and subarachnoid hemorrhage.

The patient underwent gentle traction in the neutral position until repeated cranial computed tomography revealed no progression of the epidural hematoma. Thereafter, the atlantoaxial dislocation was reduced by using partial odontoidectomy via a video-assisted transcervical approach and maintained with posterior polyaxial screw-rod constructs and an autograft. Neurological status improved immediately after surgery, and the patient recovered completely after 1 year.

Posterior fusion followed by closed reduction is the superior strategy for posterior atlantoaxial dislocation without odontoid fracture, according to literature. But for cases with severe neurological deficit, open reduction may be the safest choice to avoid the lethal complication of overdistraction of the spinal cord. Also, open reduction and posterior srew-rod fixation are safe and convenient strategies in dealing with traumatic posterior atlantoaxial dislocation patients with neurological deficit.

## INTRODUCTION

Upper cervical injuries are frequent and represent approximately 20% of acute cervical spine traumas.^1^ However, traumatic atlantoaxial dislocation is infrequent and commonly presents as translational dislocation, mostly representing anterior dislocation. Posterior atlantoaxial dislocation without odontoid fracture is extremely rare and often results in fatal spinal cord injury after high-velocity trauma and is commonly detected at post mortem examination.^2^ Thus far, only 12 such cases have been reported in the English literature.^3–14^ All 12 cases presented mild or no neurologic deficit, with no definite relation to upper spinal cord injury. In the present study, we describe a case of traumatic posterior atlantoaxial dislocation with incomplete quadriplegia associated with a spinal cord injury due to high-velocity trauma. A review of the literature pertaining to this unusual injury is also presented.

### Informed Consent

Ethical approval was obtained from the ethics committee of Tongji Hospital. Written informed consent was obtained from the patient for publication of this case report and any accompanying images.

## CASE REPORT

A 54-year-old man was admitted to our hospital 6 hours after being in a traffic accident. His head had been struck by an automobile, and he had been referred to a local hospital with full consciousness. According to the medical record of the local outpatient clinic, the patient complained of headache, and considerable pain and stiffness in the neck. His vital signs were normal, but the neurological examinations showed obvious neurological deficits in his lower extremities with a grade 3/5 power. The initial imaging study revealed temporal epidural hematoma, subarachnoid hemorrhage, and posterior atlantoaxial dislocation. Therefore, he was transferred to the traumatology department at our hospital for definitive treatment.

**FIGURE 1:**
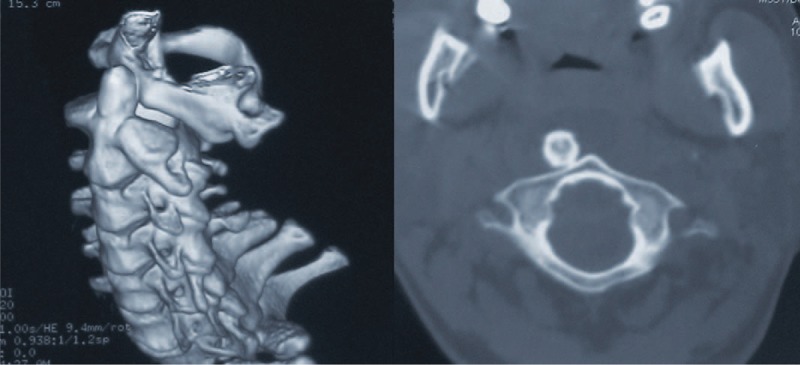
Axial CT scans and 3D reconstructions revealed intact odontoid process lied ventral to the anterior arch of atlas. 3D = three-dimensional, CT = computed tomography.

**FIGURE 2:**
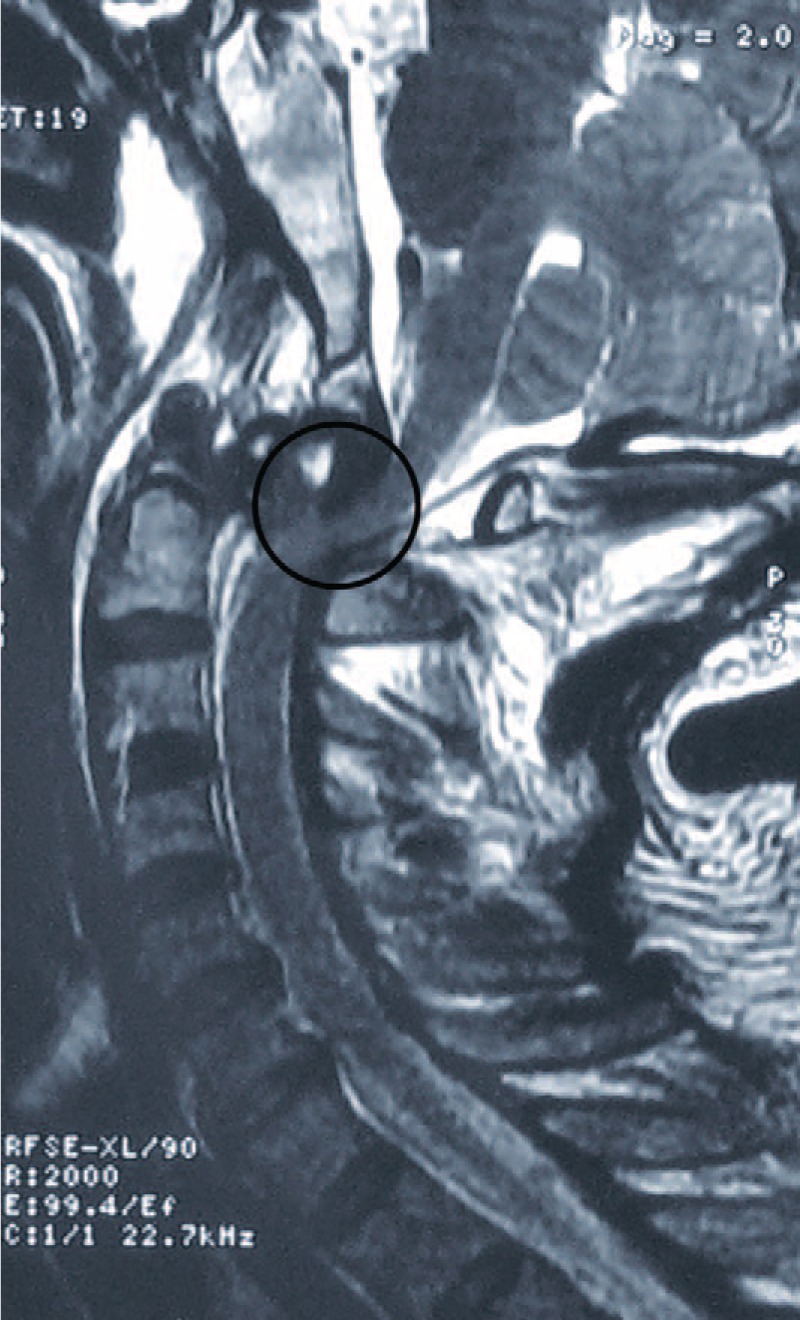
MRI showed obvious spinal cord displacement with devious course. The obliteration of the subarachnoid space and inconspicuously mild cord compression (the black circle) are demonstrated at the level of atlantoaxial dislocation. MRI = magnetic resonance imaging.

A secondary, thorough physical examination at the time of his admission revealed forehead laceration and obvious neck motion restriction in all directions. He was fully conscious and oriented with stable vital parameters. Neurological examination demonstrated grade 4/5 power in the upper limbs and grade 3/5 power in the lower limbs, with an increased patellar reflex. His Japanese Orthopedic Association (Table 1) (JOA) score was 7/17. Further cervical computed tomography (CT) with three-dimensional (3D) reconstruction showed complete posterior dislocation of the atlas, with respect to the axis, with no evidence of odontoid fracture. A skipped fracture in the lower cervical spine was also absent (Fig. 1). Magnetic resonance imaging (MRI) of the cervical spine demonstrated inconspicuously mild cord compression, with obvious spinal cord displacement and obliteration of the subarachnoid space at the level of the atlantoaxial dislocation. On T2-weighted MRI, no abnormal intramedullary signal images of the cervical spine were noted. However, retropharyngeal hematoma and edema were found at the C4 level (Fig. 2).

**TABLE 1:**
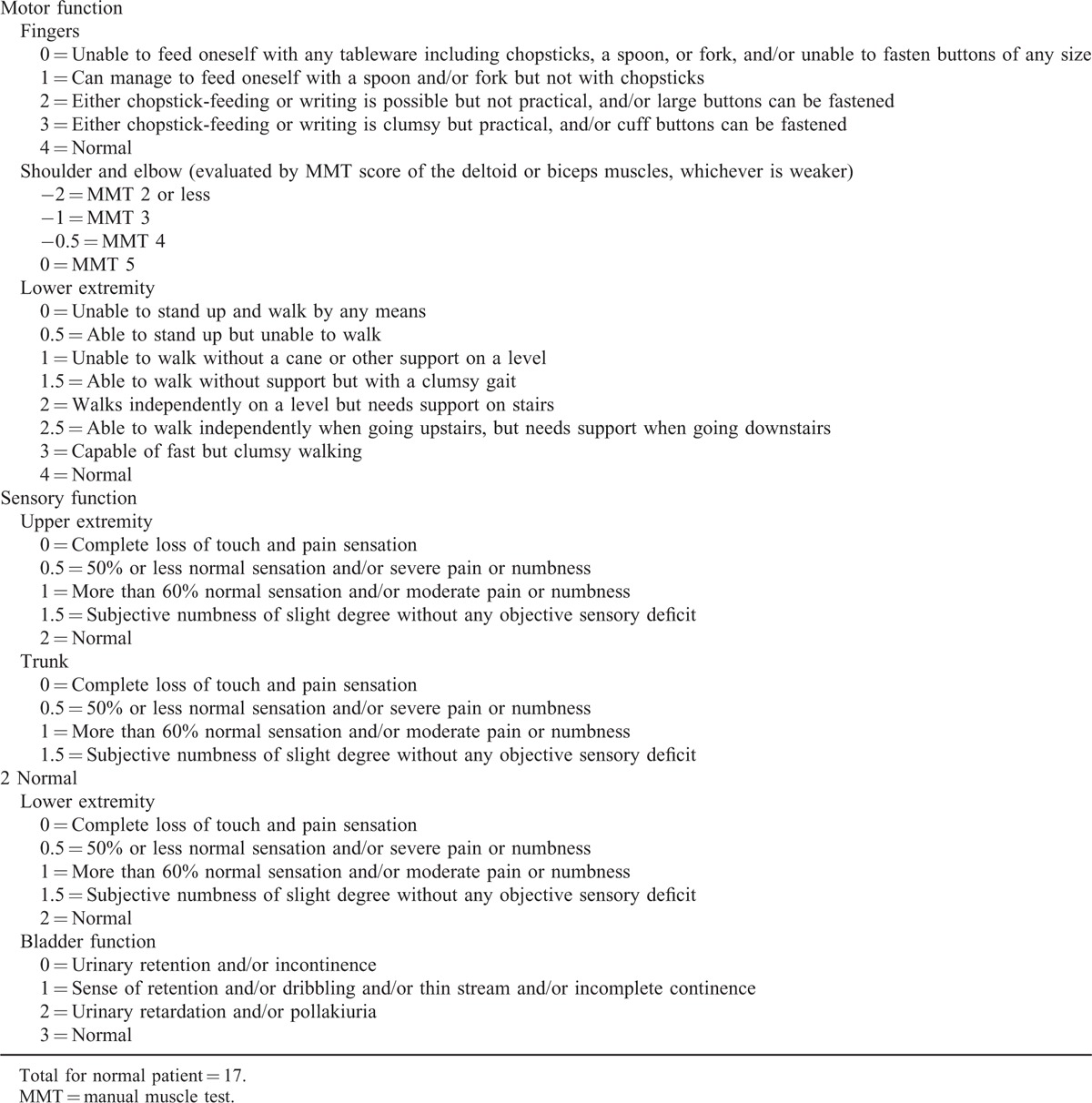
Japanese Orthopaedic Association Scoring System (17–2) for Cervical Myelopathy

**FIGURE 3:**
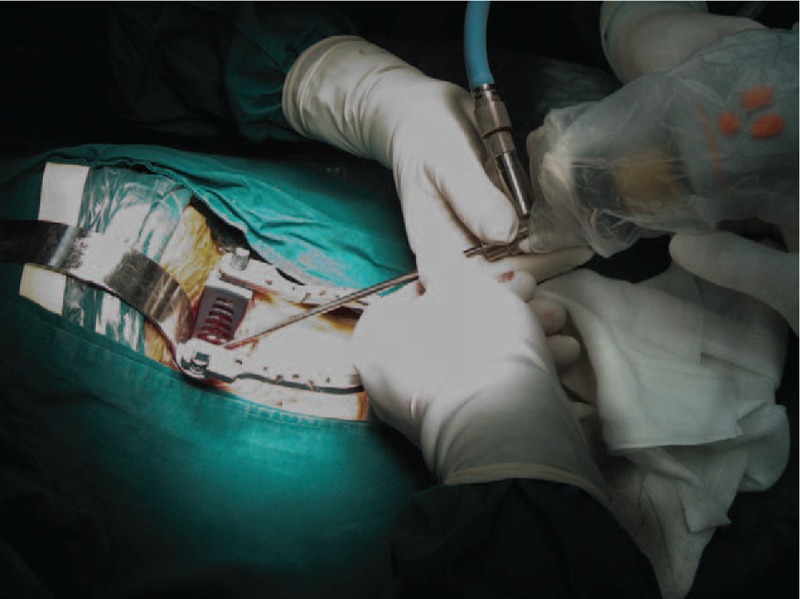
An endoscope for arthroscopy was introduced to the operating field for visualization.

Cervical traction was applied in the neutral position using a 4-kg weight during immobilization. The patient remained neurologically stable throughout the preoperative period. On hospital day 4, the patient was transferred to our department for surgery after repeated cranial CT scans confirmed no progression in the temporal epidural hematoma with conservative treatment. Closed reduction was not obtained with 4-kg traction under general anesthesia. Considering the relatively severe neurologic impairment of the patient, open reduction was conducted to avoid neurological deterioration resulting from further closed reduction attempts with gradual manual skull traction. First, the ventral aspect of the C1–C2 complex was exposed via a video-assisted transcervical approach technique.^15^ Briefly, a small transverse incision was made at approximately the C3–C4 level. Via the standard Smith–Robinson approach, the locked atlas was exposed with the aid of a Caspar retractor and an S-shaped handheld retractor, which is used to maintain the mandible cephalad. An endoscope for arthroscopy was then introduced for visualization (Fig. 3). Partial odontoidectomy was performed to remove all of the bone fragments impeding the reduction of the atlas. The resection proceeded in a “top-down” fashion using an eggshell technique with an electrical burr and Kerrison rongeur. Thereafter, only partial reduction was obtained, even with the aid of traction. Anterior fusion using a transarticular screw technique was not considered. Next, anatomic reduction was successfully gained, and posterior C1–C2 fusion was performed using a polyaxial screw/rod system with the Harms technique during the same anesthesia period.

**FIGURE 4:**
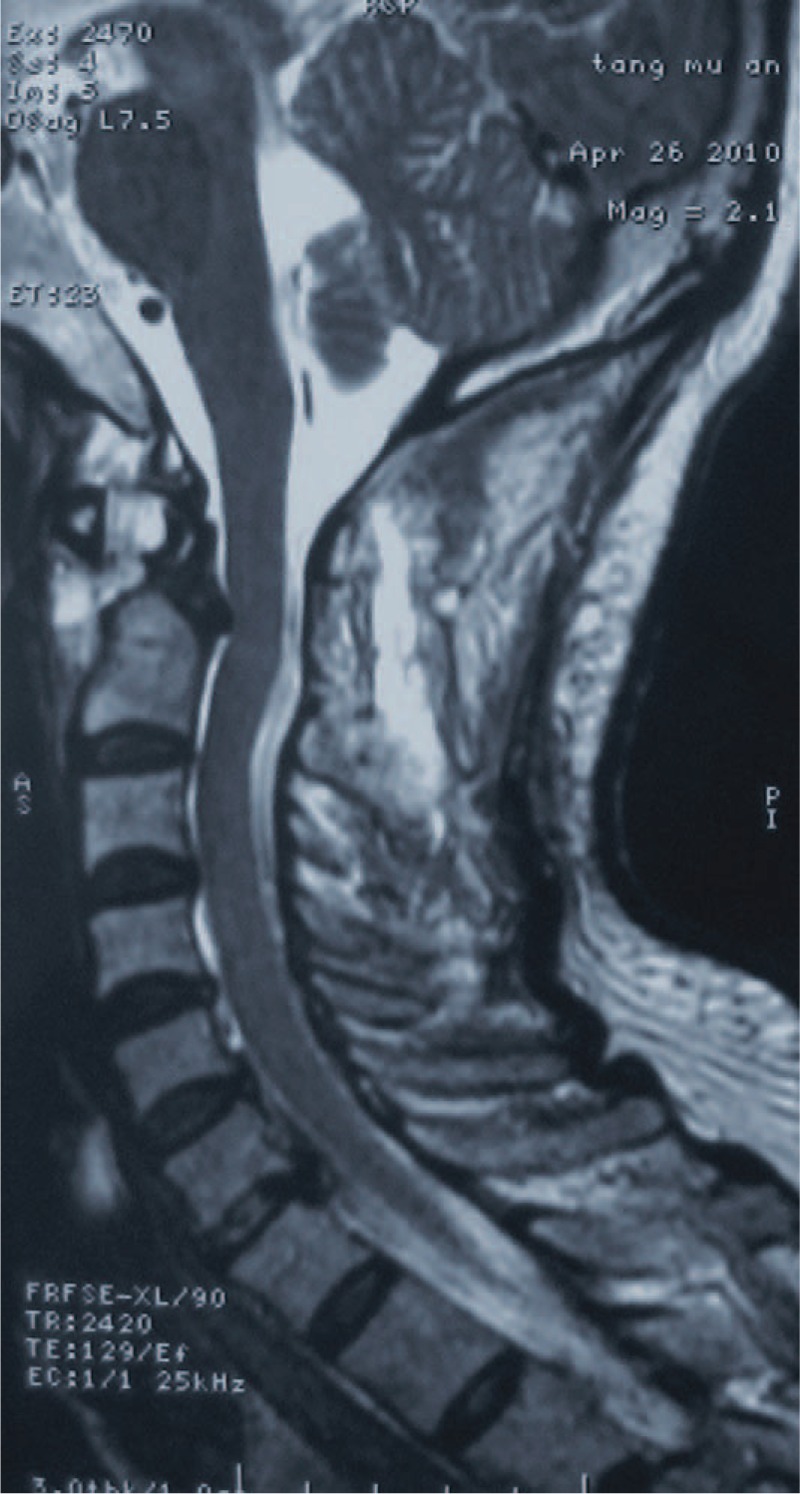
Postoperative sagittal MRI confirmed an anatomical reduction of the atlantoaxial complex and a normal spinal cord course without compression. MRI = magnetic resonance imaging.

**FIGURE 5:**
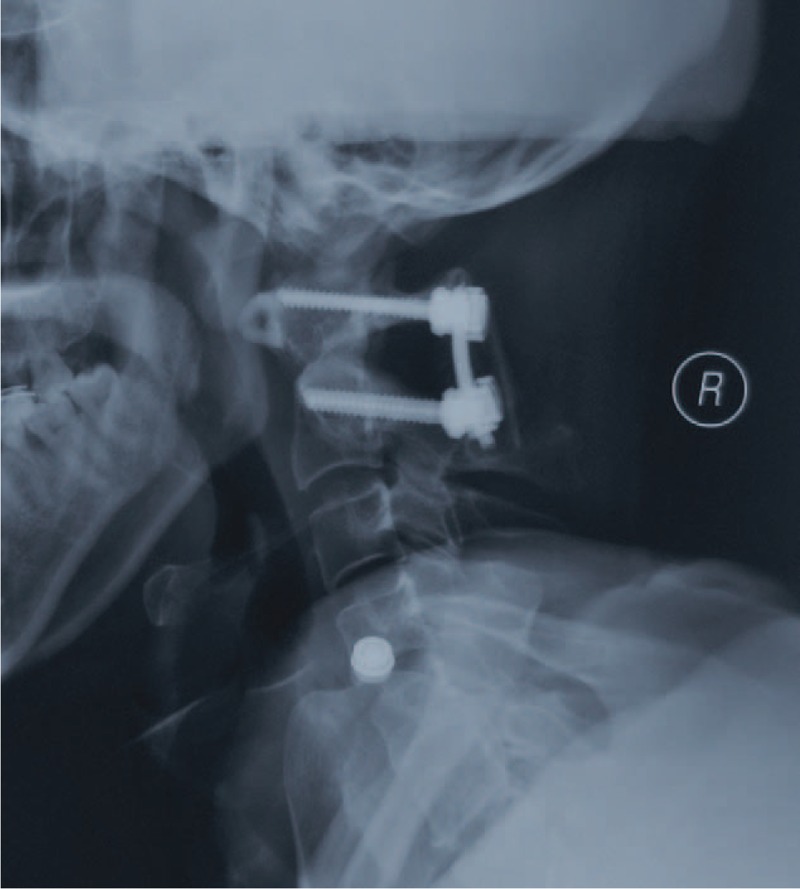
Postoperative lateral radiography showed anatomic alignment of the atlantoaxial complex with posterior polyaxial screw-rod fixation.

**FIGURE 6:**
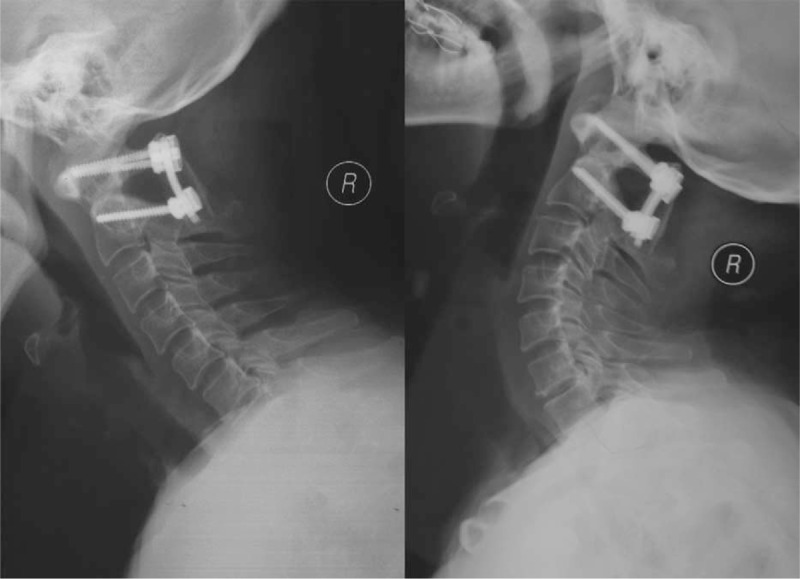
Dynamic lateral X-ray demonstrated stability of the atlantoaxial complex with solid autograft fusion at 6 months postoperation follow-up.

Postoperative MRI and X-rays confirmed anatomical reduction of the atlantoaxial complex and a normal spinal cord course without compression (Figs. 4 and 5). The patient's neurological deficits gradually improved, and he was able to walk with assistance when he was discharged. No postoperative complication was noted. A cervicothoracic brace was applied for 3 months. At the 6-month postoperative follow-up, a solid fusion was evident on dynamic plain film and the patient's JOA score had improved to 14/17 (Fig. 6). At the 1-year follow-up, the patient was neurologically intact.

## DISCUSSION

The primary movement of the atlantoaxial complex is axial rotation, and the flexion–extension movement is quite limited; additionally, the sagittal translation between the atlas and axis is also restricted to 3 mm at most in adults.^16^ Only the following 2 circumstances exist under which posterior atlantoaxial dislocation can occur: when the odontoid process is fractured or when the anterior arch of the atlas slips over the tip of the odontoid with disruption of all critical atlantoaxial ligaments, except the transverse ligament.^17^ Because the odontoid is angled posteriorly, a mean of 13° makes such a posterior slip possible in anatomy.^18^ It has been proposed that hyperextension with variable amounts of distraction may be the probable mechanism of posterior dislocation without the odontoid fracture.^5^ This latter mechanism seems to be applicable in our patient and most reported cases in which facial or neck injuries have commonly co-occurred. According to an anatomical study in the Chinese population, the average height of the anterior tubercle of the atlas is 10.23 ± 1.32 mm, the average odontoid process height is 15.25 ± 2.11 mm, and the average retroversion angle of the odontoid process is 12.23 ± 4.27.^19^ The corresponding data in our patient were 9.1 mm, 16.2 mm, and 19.1°, respectively, which are in the normal range. However, the retroversion angle of the dens was close to the normal maximum range, a finding that may be the anatomical basis of the posterior dislocation without fracture in our patient.

What distinguishes our case from previous ones are the extent of the neurological deficits, associated injuries, and the treatment strategy adopted for these neurological deficits and injuries. Three approaches are typically applied: closed reduction without fusion, closed reduction with fusion, and open reduction with fusion. Cervical traction was applied in the first case reported by Haralson and Boyd^5^ with successful reduction, consequently becoming the first-line approach to treat subsequent similar patients.^6,7^ However, residual instability and incomplete reduction have been observed after closed reduction.^10^ Posterior fusion with cervical wiring after closed reduction was accordingly applied in 3 cases to avoid residual instability or incomplete reduction.^5,9,10^ Although the closed reduction procedure is relatively safe when the patient is awake and alert, Sud et al^11^ reported a patient developing quadriparesis during traction, indicating that the procedure is potentially dangerous, and the technically demanding nature cannot beunderestimated. Open reduction is therefore the choice for failed closed reduction cases or to avoid the lethal complication of overdistraction of the spinal cord.^8,11–14^

In our case, we adopted open reduction without attempting closed reduction based on the severity of the neurologic impairment, extent of displacement of the bone structure, and distortion of the spinal cord at the dislocation level. The previously reported cases that underwent closed reduction were all neurologically intact except for 2 cases with mild abnormal sensation and mild motor function impairment in the left upper extremity. In our case, more severe neurological function loss was observed, including grade 4^+^/5 power in the upper limbs and grade 3/5 power in the lower limbs, with an increased patellar reflex, and MRI showed an obliterated subdural space and even mild spinal cord compression. Considering the loss of “neurological function reserve” and “anatomical free space reserve” in our patient, there was less possibility for him to endure temporary overdistraction or more spinal canal encroachment during closed reduction, which can lead to neurological deterioration. Partial odontoidectomy directly removes the obstacle for reduction and causes less spinal cord harassment. We introduced the video-assisted transcervical approach to address various pathological conditions ventral to the craniocervical junction because of its obvious merits.^15^ First, this technique significantly reduces approach-related morbidity and mortality compared with the transoral approach. Second, it is a less invasive procedure because it needs less dissection for exposure and the aid of endoscopic visualization avoids damage to important anatomic structures.

We found that the disrupted atlantoaxial complex could not maintain anatomical reduction under traction alone after partial ondontoidectomy, and a lever arm needed to be applied between the axis and atlas to achieve anatomic alignment. Instrumentation with fusion was also mandatory. We chose the posterior polyaxial screw-rod technique over posterior wiring and transarticular screw techniques based on the following advantages: first, posterior wiring techniques cannot provide satisfactory stability in axial rotation and lateral bending; the techniques also have limitations in resisting translational shear, which was demonstrated by incomplete translational reduction on postoperative lateral films in the cases of Fox and Jerez^8^ and Zhen et al.^13^ Second, anatomical reduction should be obtained and maintained before and throughout transarticular screw placement. However, insertion of pedicle screws in C1 and C2 is independent of the reduction maneuver; furthermore, the screws can serve as points of leverage, facilitating reduction with great convenience. Third, the trajectory of the C2 pedicle screw is more medial than the transarticular screw, thus inherently reducing the risk of vertebral artery violation. Fourth, the polyaxial screw-rod construct alone can provide the greatest biomechanical stability.

## CONCLUSIONS

Posterior atlantoaxial dislocation without odontoid fracture is extremely rare, and all of the cases, according to the reported literature, were mild or having no neurologic deficit. Additionally, posterior fusion followed by closed reduction is the superior strategy. But for cases with severe neurological deficit, open reduction may be the safest choice to avoid the lethal complication of overdistraction of the spinal cord. Therefore, open reduction and posterior srew-rod fixation are safe and convenient strategies in dealing with traumatic posterior atlantoaxial dislocation patients with neurological deficit.
